# Consolidation of the optoelectronic properties of CH_3_NH_3_PbBr_3_ perovskite single crystals

**DOI:** 10.1038/s41467-017-00567-8

**Published:** 2017-09-19

**Authors:** Bernard Wenger, Pabitra K. Nayak, Xiaoming Wen, Sameer V. Kesava, Nakita K. Noel, Henry J. Snaith

**Affiliations:** 1 Clarendon Laboratory, University of Oxford, Parks Road, Oxford, OX1 3PU UK; 2 Australian Centre for Advanced Photovoltaics, University of New South Wales, Sydney, 2052 New South Wales Australia; 3 Centre for Micro-Photonics, Swinburne University of Technology, Hawthorn, Victoria 3122 Australia

## Abstract

Ultralow trap densities, exceptional optical and electronic properties have been reported for lead halide perovskites single crystals; however, ambiguities in basic properties, such as the band gap, and the electronic defect densities in the bulk and at the surface prevail. Here, we synthesize single crystals of methylammonium lead bromide (CH_3_NH_3_PbBr_3_), characterise the optical absorption and photoluminescence and show that the optical properties of single crystals are almost identical to those of polycrystalline thin films. We observe significantly longer lifetimes and show that carrier diffusion plays a substantial role in the photoluminescence decay. Contrary to many reports, we determine that the trap density in CH_3_NH_3_PbBr_3_ perovskite single crystals is 10^15^ cm^−3^_,_ only one order of magnitude lower than in the thin films. Our enhanced understanding of optical properties and recombination processes elucidates ambiguities in earlier reports, and highlights the discrepancies in the estimation of trap densities from electronic and optical methods.

## Abstract (web summary)

Metal halide perovskites for optoelectronic devices have been extensively studied in two forms: single-crystals or polycrystalline thin films. Using spectroscopic approaches, Wenger et al. show that polycrystalline thin films possess similar optoelectronic properties to single crystals.

## Introduction

Since 2012, there has been increasing interest in lead halide perovskites, due to their outstanding performance as semiconductors used in optoelectronic applications such as photovoltaic cells^1–3^, light-emitting diodes^4^ and optically pumped lasers^5, 6^. In such devices, the perovskite thin films are deposited through vapour phase^7^ or solution deposition processes^2^. The combination of high hole and electron mobilities^8^, low exciton binding energies^9, 10^, small effective masses^10^, large light absorption coefficients^11^ and long carrier lifetimes and diffusion lengths^12^, forms the basis for the high efficiency of optoelectronic devices fabricated from these materials. However, recent reports indicate that the full potential of metal halide perovskite has not yet been reached. For example, spatial heterogeneity in the photoluminescence (PL) efficiency^13^, the orientation of the polycrystalline grains^14^, and ‘light healing’ effects^15–17^ illustrate that defects are still present and that there remains scope to further improve the thin films. In polycrystalline materials, defects are expected to be most prominent at the top and bottom surfaces and at grain boundaries. Moreover, with diffusion lengths on the same order of magnitude as the grain size^12^, surface defects are likely to be readily populated, and thus the overall properties of the materials should be governed by surface properties. For these reasons, several research groups have proposed to study macroscopic perovskite single crystals to distinguish the intrinsic properties of the bulk materials from their surface properties^18–22^. Several fabrication routes have been reported, and can be categorized as slow crystallization via the cooling of supersaturated solutions^23^, crystallisation by addition of anti-solvent^18^ or, in specific solvents rapid growth by temperature increase (dimethylformamide (DMF) or γ-butyrolactone (GBL))^22, 24^. In particular, single crystals of methylammonium lead halides MAPb*X*_3_ (where *X* = I, Br or Cl) have been widely investigated. Outstanding properties such as ultralow defect densities (below 10^11^ cm^−3^)^18, 25^ and extremely long carrier lifetimes (up to 15 μs)^26^ and diffusion lengths (above 175 μm)^25, 27^ have been inferred from combinations of optical and electronic measurements. Interestingly, despite the apparent high quality of most of the single crystals investigated, there is a wide disparity in the optical properties (i.e., absorption and photoluminescence) reported from the same materials. In early 2015, Shi et al^18^. and Dong et al^27^. simultaneously reported exceptional properties of perovskite single crystals with absorption edges located more than 0.1 eV lower in energy than their polycrystalline thin film counterparts. These remarkable properties were later reported by different authors, together with a large red shift of the PL emission^22, 28, 29^. Surprisingly, other groups have observed almost identical light emission and absorption properties to the thin films^30–32^. The main reason invoked to explain these differences is that with single crystals it is possible to observe bulk properties away from the surface^20, 33^, and a postulation has been made that the band gap in the bulk of the crystal is lower than the band gap near the surface or in polycrystalline thin films^20^. In addition, there currently appears to be a contradiction between the very low electronic defect densities within single crystals, as determined via electronic measurements, and the observation of a very fast decay component to the photoluminescence from such materials.

Here we investigate in detail the optical properties of single crystals of MAPbBr_3_ using light transmission spectroscopy, ellipsometry, and spatially resolved and time-resolved PL spectroscopy. We provide unambiguous evidence that the optical properties of single crystals are very similar to their polycrystalline counterparts and shed light on the reported disparities. Additionally, we provide an analytic model for understanding the PL decay kinetics in perovskite materials which includes both diffusion of carriers into the crystal, and electron trapping. This model replicates the PL dynamics, and through simplification of the model, we determine the trap density in perovskite single crystals and thin films.

## Results

### Determination of the absorption coefficient of MAPbBr_3_

**Fig. 1:**
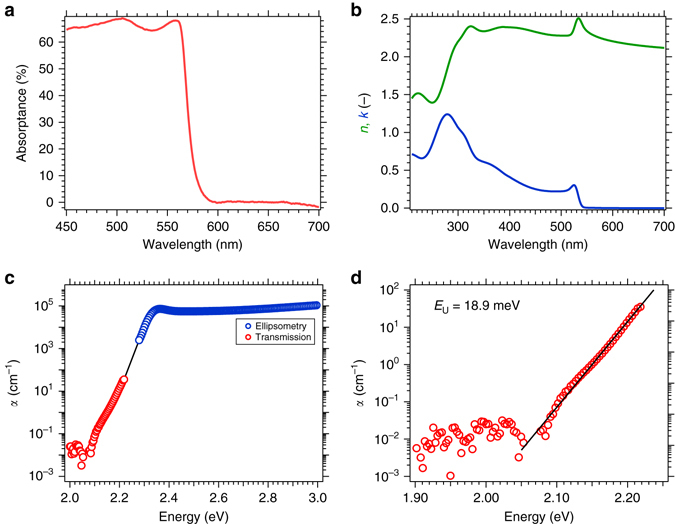
Absorption properties of a CH_3_NH_3_PbBr_3_ single crystal. **a** Absorptance spectrum calculated from total transmittance and reflectance. Below 560 nm, no light is detected in transmission mode (Supplementary Fig. 1). **b** Optical constants (*n, k*) measured by ellipsometry. **c** Absorption coefficient obtained by the combination of transmission (*red circles*) and ellipsometry (*blue circles*) data. For transmission data *α* is calculated from Beer-Lambert law (crystal thickness 2.13 mm) and for ellipsometry from the attenuation coefficient *k* (see main text). **d** Estimation of the Urbach energy by fitting the exponential tail of the absorption spectrum

We synthesized large crystals (larger than 1 cm) of MAPbBr_3_ using the rapid growth route with increasing temperature in DMF^22, 24, 34^. Despite the large thickness of such crystals (typically 2 mm) we have been able to collect optical spectra in transmission and reflection mode employing an integrating sphere, due to their good optical quality. As we show in Fig. 1a, we observe a sharp increase in the absorption at 560 nm. However, due to the large optical density of the crystals, the absorptance rapidly saturates, measured as 1 – *T*_T_ (total transmittance) −*R*_T_ (total reflectance), and can no longer be resolved for shorter wavelengths (see also Supplementary Fig. 1). The fast saturation is due to the low penetration depth of visible light into the material (*d*_1/e_ ≈ 80–180 nm). In order to characterize the optical constants over the full visible spectral range, we use spectroscopic ellipsometry on a large MAPbBr_3_ single crystal. In Fig. 1b, we show the *n* and *k* values obtained by fitting the ellipsometry data using 5 general oscillator functions: 1 Cody-Lorentz^35^, 3 Gaussian and 1 Psemi-MO^36, 37^. We give full details of the optical fitting in the Supplementary Fig. 2. In addition, our analysis reveals a surface roughness of 8.5 nm. The optical constants which we estimate from our measurements are almost identical to those reported recently by Park et al. (Supplementary Fig. 3)^32^. We observe a sharp rise in absorption at 540 nm featuring a small peak characteristic of a strong excitonic transition^30^. This is followed by a typical continuum of band absorption including several peaks. We calculate the absorption coefficient, *α*, of the crystals over the full measurement range by combining data obtained from transmission and reflection spectroscopy (*α* = −(1/*d*) ln(*T*_T_ / (1−*R*_T_) where *T*_T_ and *R*_T_ are measured in an integrating sphere and *d* the thickness of the crystal), along with ellipsometry (*α* = 4*πk*/*λ* where *k* is the attenuation coefficient and *λ* the wavelength in air). The combination of the absorption coefficient measured from ellipsometry and transmission/reflection spectroscopy is shown in Fig. 1c. Our measurement of the absorption coefficient confirms the strong absorption above the band edge characteristic of a direct bandgap material with *α* up to 10^4^–10^5^ cm^−1^. Below the excitonic transition, we observe a fast decrease of the absorption coefficient over several orders of magnitude. We note that the maximum optical density (OD < 4) that can be measured with the UV-VIS spectrometer corresponds to *α *≈ 35 cm^−1^ for this particular crystal thickness (*d* = 2.13 mm). This value of *α* is typically in the range that has been previously observed with ultra-sensitive techniques such as Fourier-transform photocurrent spectroscopy^38, 39^ or photothermal deflection spectroscopy^11, 40, 41^. This result unambiguously illustrates that when the crystals are probed with conventional UV-Vis transmission spectroscopy, it is only the sub-band gap Urbach tail which is being probed. We estimate the optical band gap of these crystals by performing Tauc analysis of the combined ellipsometric and transmission absorption spectra, which we show in Supplementary Fig. 4. We determine a Tauc band gap of 2.31 eV, in close agreement with that determined from polycrystalline thin films of the same material^41–43^. Although the Tauc analysis enables easy comparison between materials, we do note that the Tauc analysis for this material underestimates the bandgap due to the presence of an excitonic transition. Therefore, we fit the absorption spectrum using Elliott’s model^44^ and determine a bandgap of 2.39 eV with an excitonic binding energy of 36 meV (Supplementary Fig. 5). This compares well with previously published values^30, 43^, and is also almost equivalent to the values measured for thin films (Supplementary Fig. 5). We note that the binding energy is higher than that estimated with more accurate techniques such as magneto-optical spectroscopy^45^. Interestingly, since the UV-Vis transmission spectroscopy probes the Urbach tail, it is possible to estimate the energetic disorder in the semiconductor by fitting this data with an exponential function (see Fig. 1d). We determine an Urbach energy of 19 meV, which is within the range of that previously estimated in polycrystalline thin films of MAPbBr_3_, which have varied from 17 to 23 meV^40, 41^. This indicates that the intrinsic electronic disorder in these single crystals is comparable with an average polycrystalline thin film^46^.

In principle, we expect sub band gap absorptions from trap states to be discrete from the Urbach tail and emerge at low energy, with the strength of the light absorption being proportional to the trap site density. Unfortunately, however, our crystals do scatter quite a significant amount of light, and even though we are employing an integrating sphere, part of the light-scattered forward into the crystal, or diffusely reflected back into the crystal from the rear surface will not be detected in our set up. Hence, our sensitivity limit, appears to be *α *≈ 0.1 cm^−1^. This is slightly lower than the best sensitivity for PDS of thin films. However, we cannot claim from our absorption measurements here, that the defect density of our single crystals is any lower than that observed in polycrystalline thin films.

### Photoluminescence properties and emission filtering

**Fig. 2:**
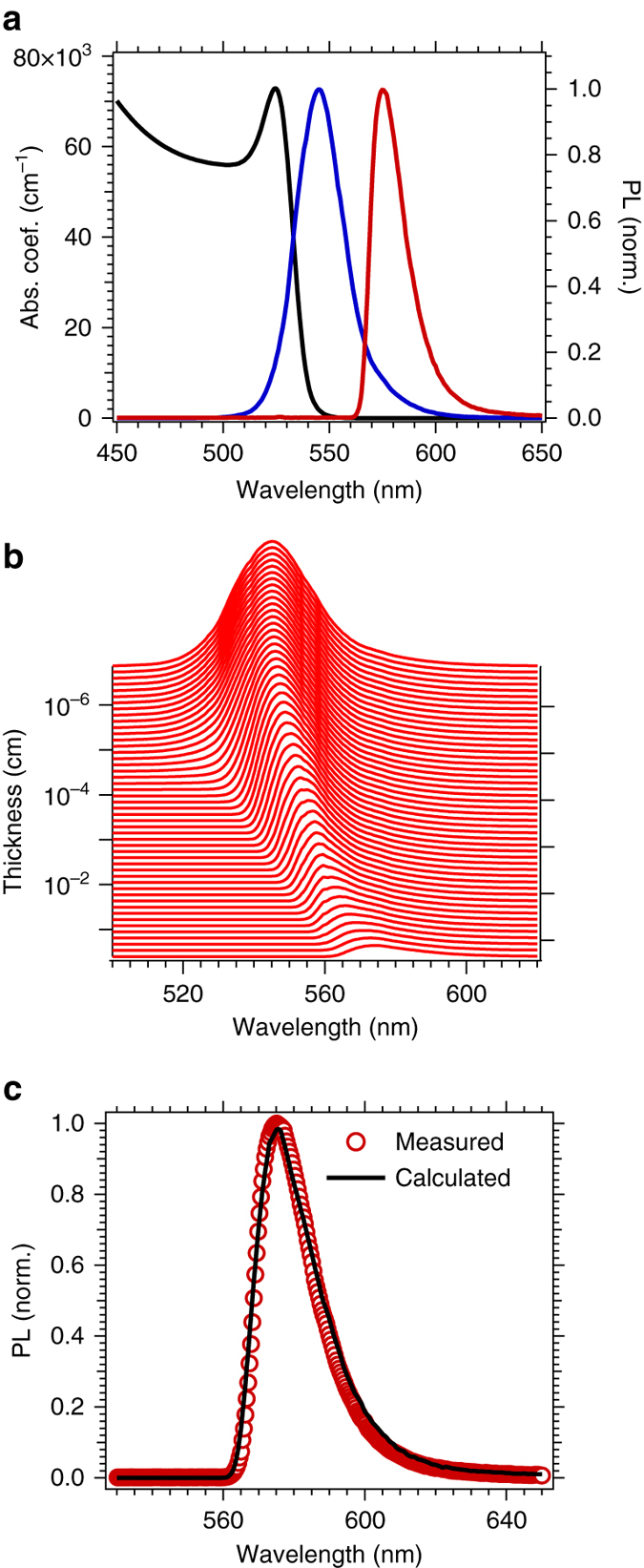
Photoluminescence (PL) of single crystals of CH_3_NH_3_PbBr_3_. **a** Absorption coefficient (see Fig. 1c) and PL emission spectrum collected from the front (*blue*) and from the rear (*red*) of the crystal (Supplementary Fig. 6). **b** Calculated attenuation and shift of the PL emission through the thickness of a 2 mm thick crystal by reabsorption. The initial PL spectrum (*d* = 0 mm) is the one obtained from the front of the crystal (*blue curve* in **a**). **c** Comparison of the normalised PL spectra measured from the rear of the crystal (*red circles*) and that calculated from reabsorption within the bulk of the crystal (last spectrum of b, with *d* = 2.13 mm)

Another ambiguity in the current literature is the position of the photoluminescence peak. Some reports display the PL peak at lower energy than the Urbach tail^18, 22, 47^, others show it to be near the above band gap absorption onset^25, 27, 48, 49^ To understand these discrepancies, here we measure the steady-state photoluminescence in two different configurations: front illumination where the emitted light is collected from the same side as the excitation spot and back illumination where the sample is excited from the back side and the emitted light travels through the thickness of the crystal before being detected (Supplementary Fig. 6). With front illumination, we observe a single PL peak, centred at 545 nm with a slight asymmetric tail extending towards longer wavelengths. When the light is collected from the opposite side of the crystal, we find that the spectrum is markedly red-shifted with a maximum at 575 nm and a very sharp rise on the blue edge. We show both PL spectra, along with the above bandgap absorption spectra in Fig. 2a. We attribute this shift in the PL to lower energy to be due to the filtering of the PL via reabsorption within the crystal as the light propagates throughout the thickness of the crystal. Capitalizing upon the accurate absorption coefficient we have measured from ellipsometry and transmission experiments, we calculate the effect of reabsorption through the thickness of the crystal (*d* = 2.13 mm) using Beer–Lambert absorption *I*_z, λ_ = *I*_0, λ_exp(−*α*_λ_ *z*) where *I*_z, λ_ is the radiant intensity after a distance *z* from the surface and *α*_λ_ the napierian absorption coefficient of MAPbBr_3_ (Fig. 2b, c). Starting with *I*_0, λ_ as the emission spectrum collected with front illumination, we observe that the emission spectrum which we calculate is continuously red-shifted and depleted as the light travels through the crystal. In surprisingly good agreement, we verify that our calculated spectrum obtained for PL travelling through the thickness of the entire crystal corresponds precisely to the spectrum we measure from the rear of the crystal. We conclude, therefore, that re-absorption of internally emitted light is the main reason for the spectral shift observed in single-crystal perovskite and that there is no significant change in the bandgap of the material, which corrects previous misinterpretations^18, 20^.

As an aside, we note that we have not had to include the influence of photon-recycling^46^ for these macroscopic crystals of MAPbBr_3_. In the process of photon-recycling, the PL is reabsorbed, generates more charges, and the recombination events of these charges lead to reemission of light. There could be a number of reasons why we do not need to include photon-recycling here, even if photon-recycling is happening multiple times in the bulk of the crystal. One key issue is the fact that at each recycling event, the light is emitted isotropically. Therefore, only a small fraction of the reemitted light will be propagating along the same path as the light travelling across the thickness of the crystal. In addition, all photons emitted outside a small angular cone will undergo total internal reflection when reaching the far side of the crystal and will thus not contribute to the detected emission. This is in agreement with the recent reports of low external emission due to photon recycling (around 0.5 %) in single crystals^50^ and a relatively modest (with respect to the thickness of the crystal) photon recycling effective length (*L*_PR_ = 4.2 µm)^51^. However, we emphasise that this does not imply that photon recycling is not an influencing factor in the optoelectronic properties of thin films.

### Depth profiling with two-photon excitation PL spectroscopy

**Fig. 3:**
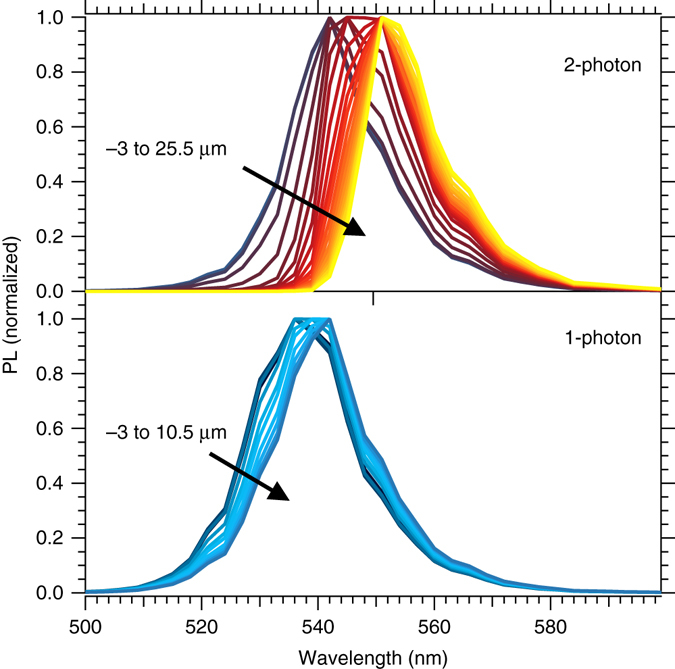
Photoluminescence (PL) spectra with 1-photon (1P) and 2-photon (2P) excitation. (*top*) Normalized PL spectra for 2P excitation (*λ*_exc_ = 800 nm) where the focal depth is varied from −3 (above the surface) to 25.5 µm. (*bottom*) Normalized PL spectra for 1P excitation (*λ*_exc_ = 488 nm) where the focal depth is varied from −3 (above the surface) to 10.5 µm

Two-photon PL spectroscopy has also been performed on perovskite single crystals^22, 29, 52, 53^, and usually the two-photon PL decay kinetics are slower than the 1-photon kinetics. Here, we performed PL spectroscopy in confocal geometry with two-photon (2P) excitation. In this configuration, the emission region can be profiled by focusing the excitation beam below the crystal surface. As two-photon absorption increases quadratically with the excitation density, the material is excited solely near the focal spot. We excite the sample using near infrared laser light with energy below the band edge (*λ*_exc_ = 800 nm) so that one-photon absorption is negligible. We obtained the PL spectra by increasing the focal depth from −3 μm (above the surface) to 25.5 μm. As we show in Fig. 3, we observe a red shift of the emission from 540 to 550 nm. When the sample is excited above bandgap (i.e., 1-photon (1P) excitation) we also observe a slight shift but over a much shorter range than for 2P excitation. We observe not only a shift in the emission maximum for 2P PL, but also that the asymmetry of the peak increases. This observation is consistent with re-absorption of light emitted from inside the crystal in a similar manner to what we observed for the PL emission collected through the thickness of the crystal (Fig. 2). This effect has been previously reported by Yamada et al^52, 53^. who also interpreted the red shift in the 2P PL spectrum as being due to reabsorption of emission generated within the bulk of the crystal. However, with similar experiments, Wu et al^20^ concluded that this shift indicated a different bandgap between the surface and the bulk of the material, in line with the reported observations of a single low energy emission^18, 22^. Our experiments clearly show that the peak shift and the low energy are consistent with re-absorption of light emitted from within the crystal and not with a change of the bandgap. Our interpretation is also consistent with lasing experiments performed under 2P excitation in microlasers where a 6 nm shift (539–545 nm) was observed and optical gain occurred slightly below the band edge^6^.

### Time-resolved PL decays and kinetic model

**Fig. 4:**
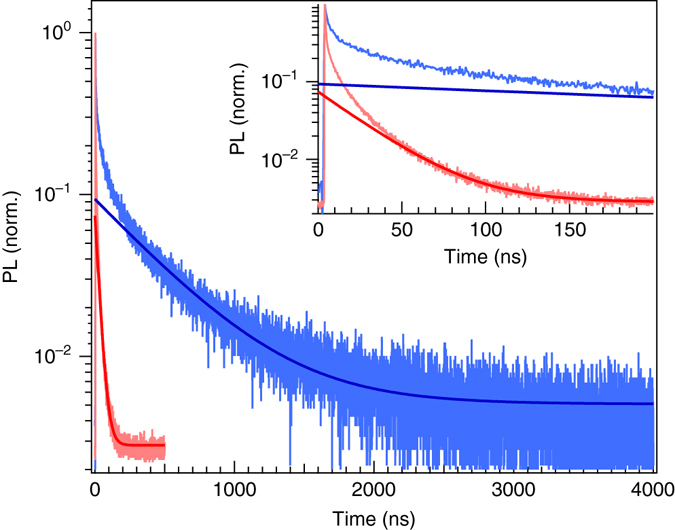
Normalized photoluminescence (PL) decays for CH_3_NH_3_PbBr_3_. Thin film (*red*) and single crystal (*blue*) excited at 447 nm. The tails of the decay are fitted with an exponential decay function with constants *k*_SC_ = 2.1 × 10^6^ s^−1^ and *k*_film_ = 3.6 × 10^7^ s^−1^. *Inset*: zoom on shorter time scale

A further discrepancy in the literature is the PL decay lifetime reported for perovskite single crystals: PL decay is often used as an assay to indicate the optoelectronic quality of the crystals, where fast PL decay is usually interpreted to stem from fast non-radiative decay pathways, indicative of a high density of electronic trap sites through which recombination can occur^18, 25, 26, 30^. In Fig. 4, we show the PL decays of single crystals and polycrystalline thin films of MAPbBr_3_. For both samples, we observe an initial fast decay phase which is followed by a slower phase. For the single crystal, the slow phase extends over several microseconds whereas no PL is observed after 200 ns for the thin film. This qualitatively suggests that the defect density in the single crystals is significantly lower than for the thin films. In order to gain quantitative information about the quality of the material we fit the tail of the PL decays with a single exponential function. We obtain a good fit quality and determine recombination constants of *k*_SC_ = 2.1 × 10^6^ s^−1^ (*τ*_SC_ ≈ 480 ns) and *k*_film_ = 3.6 × 10^7^ s^−1^ (*τ*_film_ ≈ 28 ns) for the single crystal and the thin film, respectively. This suggests that the defect density is about one order of magnitude higher in thin films (see detailed discussion below). However, we do observe that the fast decay phase is always present in the single crystals (see Supplementary Fig. 7). Notably, the fast decay rate does not strongly depend on excitation fluence, and is even present at the lowest fluences for which we could obtain good quality data which correspond to an initial carrier density of about 10^15^ cm^−3^. Therefore, it is unlikely that this fast phase is dominated by bimolecular radiative recombination of free carriers, since this would be expected to speed up with increasing fluence.

These observations either imply that there is a fast non-radiative decay in single crystals, or that there are other mechanisms at play which have not previously been considered. In order to determine the overall fraction of radiative vs. non-radiative decay, we measure the PL quantum efficiency (PLQE) for a single crystal by placing it in an integrating sphere and irradiating it with 405 nm illumination of 9 mW intensity (illumination area ~ 0.1 mm^2^) and measuring the emission. As we show in Supplementary Fig. 8 the emitted spectrum from the single crystal is a combination of surface emission (characteristic band gap emission centred on 547 nm), with a majority contribution from attenuated emission at longer wavelengths. The overall external PLQE which we estimate is 6.4%, which, as far as we are aware, is the highest PLQE reported to date for MAPbBr_3_ singe crystal. However, single we know precisely how PL is attenuated as it passes through the crystal, we can make an estimation of the original internal PL spectrum and intensity responsible for this external long wavelength emission. By performing a reverse calculation to that which we presented in Fig. 2b, we re-estimate the original PL spectrum and intensity responsible for this long wavelength emission, and estimate an internal PLQE of 67 %. We give precise details of how we have made this calculation in the Supplementary Discussion. This indicates a very high quality crystal, and we can therefore conclude that the fast decay component which we observe in the early time decay of the PL from the perovskite single crystals does not originate from fast non-radiative recombination, otherwise the internal PLQE would be very low.

**Fig. 5:**
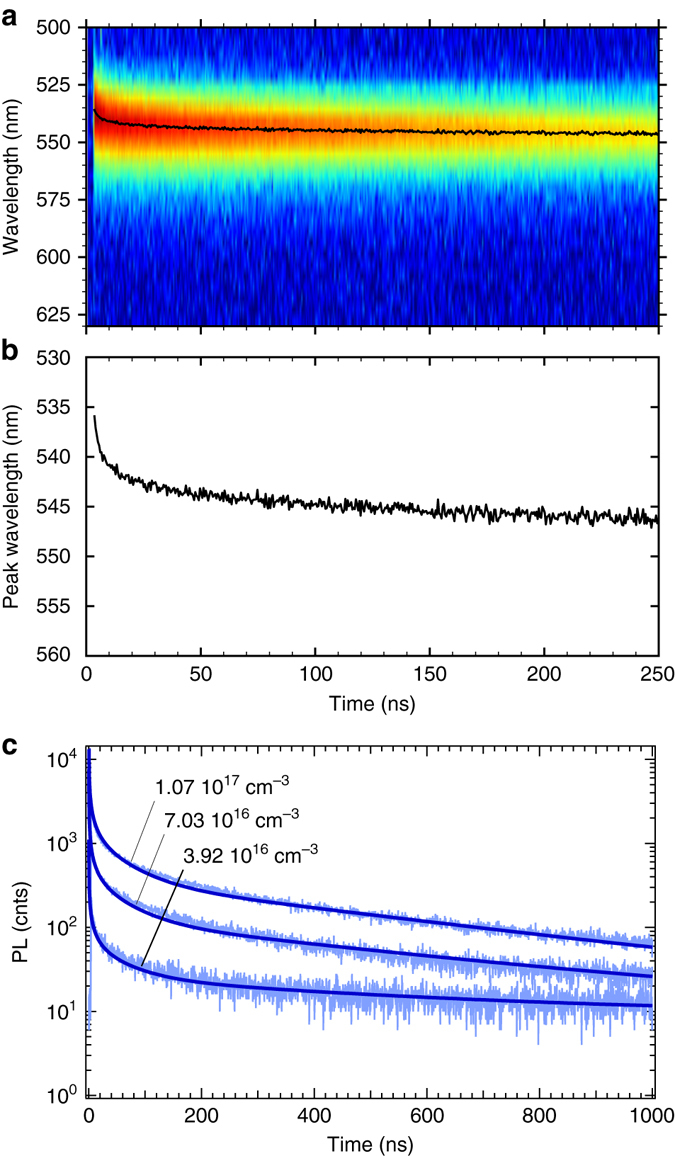
Time-resolved photoluminescence spectra of CH_3_NH_3_PbBr_3_ single crystals. **a** 2D spectral map, **b** shift of the peak centre position with time. **c** Numerical evaluation of Eq. (2) for three decays extracted from Supplementary Fig. 7. The initial carrier densities are indicated on the Figure. Parameters: *µ*_n_ = 10 cm^2^ V^−1^ s^−1^, *µ*_p_ = 50 cm^2^ V^−1^ s^−1^, *k*_2_ = 1×10^−10^ cm^3^ s^−1^, *k*_1,n_ = 9×10^5^ s^−1^, *k*_1,p_ = 1×10^3^ s^−1^. See details of simulation in the Supplementary Discussion

To extract quantitative information about the different recombination processes we try to apply a kinetic model previously developed to describe PL decays in lead halide perovskites^16, 54^. However, as already observed for the iodide containing perovskite (MAPbI_3_)^34^, this model is unable to explain the fast decay phase observed in single crystals, although it fits the slow phase well, which we show in Supplementary Fig. 9. The principal assumption of this model is the presence of an excess of positive charges due to the preferential, long-lived, trapping of electrons. However, in this model, the trapped electrons are not in equilibrium with the conduction band population, and eventually recombine non-radiatively at longer times. We note that the early decay does not follow a monoexponential law, which indicates that the kinetics are not determined by a trap-assisted mechanism (Shockley-Read-Hall), neither are they determined by a monomolecular recombination to a sea of background carriers. We do not observe a significant speeding up of the fast component with increasing excitation intensity (as we show in Supplementary Fig. 7), which would be expected if it was primarily governed by the bimolecular recombination of free electrons and holes. However, it could be consistent with a diffusion-like process, since the carriers, although generated close to the surface, can diffuse over some distance if the non-radiative recombination rate in the bulk of the crystal is low^30, 51, 53, 55^. We measure the time-resolved emission spectra and show the 2D map in Fig. 5a. We observe a shift of the emission peak over time, which is consistent with the diffusion of carriers into the bulk of the crystal^51, 53, 55^. Similar to the filtering effect we observed with two-photon absorption, as the carriers move away from the surface, light emitted from recombination of free carriers is partially reabsorbed by the crystal.

In Fig. 4, we showed that the tails of the PL decays can be fitted with a single exponential function to determine later-time recombination constants of *k*_SC_ = 2.1 × 10^6^ s^−1^ and *k*_film_ =3.6 × 10^7^ s^−1^ for the single crystal and the thin film, respectively. To understand the meaning of the decay constant obtained from fitting the tail to the PL decay, we write the rate equations for radiative recombination in a single crystal. The intensity of the photoluminescence *I*_PL_ is proportional to the product of the electron (*n*) and hole (*p*) densities integrated over the spatial extent of the crystal. Provided the excitation spot is large as compared to the carrier diffusion length, we should be able to neglect lateral diffusion, and for simplicity, we reduce the problem to a single dimension where *z* is the distance from the surface (*z* = 0) towards the bulk of the crystal in the direction perpendicular to the surface plane of the crystal.

1 $${I_{{\rm{PL}}}}\left( t \right) \propto \mathop {\int }\nolimits n\,\left( {z,t} \right)p\left( {z,t} \right)dz \cdot $$

If we neglect Auger recombination processes, which have been shown to play a role at excitation densities slightly higher than those relevant to this work^43, 56^, we can describe the transient population of electrons by,

2 $$\frac{{\partial n\left( {z,t} \right)}}{{\partial t}} = G - {D_{\rm{n}}}\frac{{{\partial ^2}n\left( {z,t} \right)}}{{\partial {z^2}}} - {R_r} - {R_n}r,$$

where *G* is the carrier generation rate, *D*_n_ the diffusion constant, *R*_r_ and *R*_nr_ the radiative and non-radiative recombination rate. The non-radiative rate *R*_nr_ can be described with the Shockley-Read-Hall model^57^ and the radiative recombination rate as

3 $${R_{\rm{r}}} = B\left( {n\left( {z,t} \right)p\left( {z,t} \right) - n_{\rm{i}}^2} \right),$$

where *B* is the radiative recombination constant*, n(z,t)* and *p(z,t)* are the electron and hole densities respectively and *n*_i_ *is* the intrinsic carrier density. Since this model contains a number of free parameters, it is not straightforward to fit the experimental data with satisfactory confidence. However, in order to illustrate the form of the decay, we show calculated PL decays, according to Equation 2 employing sensible material parameters, along with measured PL decays in Fig. 5c. The close coincidence of the experimental and simulated curves, gives us good confidence that the presence of diffusion, bimolecular recombination and recombination to a large excess of background carriers (*p*_0_) can explain and replicate the PL dynamics.

Diffusion of carriers away from the surface will result in a rapid dilution of the carrier densities, and hence a reduction in the bimolecular recombination rate. This will influence the PL dynamics most strongly at early times, when there exists the highest concentration gradient. Experimentally, this appears to be within the first 50 ns, after which the contribution of diffusion to the depletion in carrier density will be minor. Hence, if we neglect diffusion for longer times, and considering that *n*_i_ is negligible at room temperature due to the large bandgap of MAPbBr_3_ $\left( {np - n:i^2 \approx np} \right)$, Equation (3) can be simplified as:

4 $${R_{\rm{r}}} \approx B\left( {\Delta n{\rm{ + }}{n_0}} \right)\left( {\Delta p{\rm{ + }}{p_0}} \right)$$

where Δ*n* and Δ*p* are the excess charge carrier densities generated by photoexcitation and *n*_0_ and *p*_0_ are steady-state carrier densities.

As has been previously observed, PL kinetics, photoconductivity and slow open-circuit voltage decays are consistent with the presence of an excess of free holes remaining, after the trapping of electrons into mid-gap traps with long lifetimes (µs–ms)^16, 58, 59^. Thus, we assume a quasi-steady state population *p*_*0*_ of excess holes is built up, and since Δ*n*=Δ*p*, we can further simplify equation (4) and give the full recombination rate for electrons by the following expression:

5 $${R_r} \approx B(\Delta {n^2} + \Delta n{p_{0}}).$$

As the photogenerated carrier densities decreases, the PL kinetics will become dominated by first order recombination processes occurring via radiative recombination with the excess hole population, *p*_0_. The recombination constant *k*_n_ obtained by fitting the tail of the PL decays with an exponential function is approximately equal to *Bp*_0_ and thus provides an estimation of the magnitude of the excess hole population *p*_0_. Therefore, we assume that the quasi-steady state concentration of trapped electrons gives rise to an excess hole density (*p*_0_), analogous to p-type photodoping^16, 54^.

From our observation of high internal photoluminescence quantum yield, we estimate that the majority of the available traps are occupied under such illumination conditions. Therefore, we consider that the excess hole density, which we estimate from the fit of the PL decays, is a quantitative indicator of the density of deep electron traps. In our analysis, it is explicitly the density of occupied deep traps. For a direct bandgap semiconductor, *B* is typically on the on the order of 10^−10^ cm^3^ s^−1^ ^43, 57^_,_ Thus, as mentioned above, we can determine the excess hole densities *p*_o_, which gives us an indication of the density of deep electron traps. We find trap densities of 4×10^15^ cm^−3^ for single crystals, and 7×10^16^ cm^−3^ for thin films (using a radiative recombination constant value of *B* = 5×10^−10^ cm^−3^ s^−1^ as measured by transient absorption spectroscopy)^43^. The estimated values are approximate due to the uncertainty of the *B* coefficient, the fitting procedure and the simplified model. Taking this into account we consider that electron trap densities are on the order of 10^15^ cm^−3^ in single crystals, and about an order of magnitude higher in the films (10^16^ cm^−3^). If we assume an electron mobility of 30 cm^2^ V^−1^ s^−1^, as determined for MAPbBr_3_ via electron beam induced current (EBIC)^60^, then our results are consistent with a diffusion length of 6.0 µm for single crystals, using the Einstein relationship, *D*_e_ = *μk*_*B*_ *T*/*q*, and ${L_D}{\rm{ = }}\sqrt {{D_e}\tau }$, where *D*_e_ is the diffusion coefficient, *L*_D_ is the diffusion length, *µ* the electron mobility, *k*_B_ Boltzmann’s constant, *T* the temperature, *q* the elementary charge and *τ* = 1/*k* the carrier lifetime).

We note that although it is well-known that photon recycling does influence the photoluminescence in high quality inorganic semiconductors^61, 62^, we have not implemented it here due to the small role played by this effect in large single crystals (see above).

In the thin films, the photoluminescence lifetime is substantially shorter than for the single crystals. Via fitting the tails of the decay, we reveal that the recombination rate and the deep trap density are higher in the thin films, by approximately one order of magnitude (*k*_film_ = 3.6×10^7^ s^−1^ and *N*_t,film_ ~ 10^16^) as compared to the single crystals. This results in our estimation of the diffusion length dropping to 1.5 µm for the thin films. Interestingly, we also observe a fast PL decay at early times in the thin films. We apply our model, including diffusion, to the PL decay trace of the thin film and can accurately reproduce the experimental result (Supplementary Fig. 10, Supplementary Table 1). This suggests that even for polycrystalline thin films, diffusion of carriers away from the surface and into the bulk also plays a role in the PL decay kinetics. We note that the carrier mobilities which we estimate from the fitting are lower than for the single crystal (*µ*_n_ = 1 cm^2^ V^−1^ s^−1^, *µ*_p_ = 5 cm^2^ V^−1^ s^−1^). This is consistent with an increased scattering due to higher defect density and the presence of grain boundaries, reducing the mobility in the thin films. We also note, however, that perovskite polycrystalline thin-films have been previously observed to exhibit heterogeneity in the PL decay rate from different grains^13, 63^. Therefore, although our PL decay model, which includes diffusion of carriers away from the surface, can replicate the PL decay dynamics in thin films, there may also be a fast PL decay component due to heterogeneity. In addition, the early time decay component may also be strongly influenced by fast surface recombination which we cannot separate from diffusion effects in our model.

## Discussion

In this work, we show that the long wavelength emission observed in single crystals is due to reabsorption of the emitted light within the crystal, and is not consistent with a change of bandgap in the bulk of the material. Moreover, the sharp red-shifted absorption edge often observed is due to the large optical thickness of the crystals, and the transmission measurements usually employed only being sensitive to probing the sub-band-gap tail. Although reabsorption effects on luminescence spectra and lifetime of solid-state materials are well-known and described in detail in textbooks^64, 65^, these effects have been overlooked in the recent literature on lead halide perovskites and this has led to numerous misinterpretations. For example, optical bandgaps have been substantially underestimated in many influential papers^18, 19, 22, 25–29, 66–70^. The apparent shift of the PL peak due to reabsorption has been previously reported, for example, in two-photon absorption studies^51, 53, 55^. However, several reports showed filtered emission^18, 22, 71^, sometimes blue-shifted PL^27, 72^ and in some cases double peaks^20, 71, 73^. The observation of morphological effects on the optical properties of polycrystalline films is also likely to have been biased by self-absorption effects^74, 75^. Most of these previous studies have not taken reabsorption into account, and thus provide debatable interpretations for the studied phenomena. During the review process of this article, a similar study has been reported by Fang et al^50^. who also indicate the presence of two PL peaks due to self-absorption. We note that although we have analyzed in detail MAPbBr_3_, the same filtering effect is present in MAPbI_3_ single crystals and in inhomogeneous thin films^76^. We also note that diffusion of carriers between grains in thin films, requires accounting for in spatially resolved PL measurements^63^.

Macroscopic perovskite single crystals have attracted considerable attention, in part due to the remarkably ‘superior’ properties they appear to have, as compared to polycrystalline thin films. Reported attributes include carrier diffusion lengths greater than 175 µm^25, 27^, and defect densities below 10^9^ cm^−3^. Specifically, trap densities estimated with the space-charge limited current technique have been as low as 10^8^–10^10^ cm^−3^ ^18, 22, 27, 67, 72^, and via thermal admittance spectroscopy^25^ *n*_TDOS_ about 10^7^ cm^−3^ eV^−1^, and carrier lifetimes as long as 234 µs^27^. However, the trap densities that we determine from our optical studies are orders of magnitude higher than this, but are consistent with other optical studies on perovskite crystals^20, 77^. Here, we have estimated trap densities of 10^15^ cm^−3^ for high-quality single crystal of MAPbBr_3_, which are only one order of magnitude lower than that which we estimate in thin films. Hence, there is currently disconnect between trap densities estimated via electronic contacting and probing of the perovskite crystals, and those determined via photoluminescence.

After careful consideration, it is our opinion, that the presence of mobile ionic species, which are ubiquitous in perovskite thin films^78, 79^, cannot be neglected when measuring electronic properties of single crystals. As a simple but relevant consideration here, if ionic species can diffuse throughout the crystal, then upon applying an external electric field, the ions will diffuse to and build up at the surface. This will result in a redistribution of the electric field, with the strongest field existing near the electrodes, and the externally applied electric field will be largely screened from the bulk of the crystal, similar to the situation in light emitting electrochemical cells^80^. Hence, applying analysis, which assumes uniform, or electronic space charge mediated electric fields is unlikely to reveal real information about the optoelectronic properties of the crystals.

The trap densities in perovskite thin films, typically estimated to be in the range of 10^15^–10^17^ cm^−3^ ^9, 16^, are very similar to the values which we estimate here for the bromide single crystals. We can conclude that the trap density near the surface region of the perovskite single crystals are therefore very similar to the trap density in polycrystalline thin films. We also note that the surface region in our experiments here is within a few tens of microns, since the charges diffuse over this range during the measurement window of the PL decays. In our experiments here, we only estimate a one-order of magnitude lower trap density in the single crystals to the thin films. We also note that the PL lifetimes reported from time resolved two-photon measurements on single crystals, also appear similar to the lifetimes we estimate here^55^, indicating once again that the trap density within the bulk of single crystals is only about 1–2 orders of magnitude lower than that observed in polycrystalline thin films. We further remark that if the trap density was as low as 10^−10^ cm^−2^, we would expect a purely bimolecular PL decay, with negligible contribution of charge trapping, which neither ourselves nor others have previously seen evidence for^25, 55^.

Bi et al^26^. have recently estimated a relatively low trap density (below 10^13^ cm^−3^) in MAPbI_3_ single crystals from time-resolved microwave conductivity (TRMC) measurements. However, consistent with our work here and previously, due to the long-lived deep trapping of electrons there is an excess of free holes, which explicitly have the same lifetimes as the trapped electrons. Since TRMC cannot distinguish between negative and positive carriers, it is likely that the very long-lived signal in TRMC is mainly due to the presence of these excess free holes, with a lifetime matching that of the trapped electron, which is expected to undergo non-radiative recombination. This is also consistent with PL measurements being blind to these longer lifetime decays, since only a very small fraction of the trapped electrons will undergo radiative decay via thermal repopulation of the conduction band. We also note that similarly long-lived species in TRMC experiments have also been observed in thin films of MAPbI_3_ spin-coated from a stoichiometric solution of MAI:PbI_2_ in DMF, which generally have a large density of defects^81^. With these consideration in mind, the published TRMC measurements are consistent with our observations here. In contrast to TRMC, PL measurements are explicitly sensitive to radiative recombination from the free electron population in the conduction band.

In summary, we have synthesized large single crystals of MAPbBr_3_ and measured their optical properties with various spectroscopic techniques. Combining transmission spectroscopy and ellipsometry we obtain the absorption coefficient of this perovskite crystal over six orders of magnitude. We use several techniques to characterise the photoluminescence and show that the optical properties of single crystals of MAPbBr_3_ are almost identical to the optical properties of polycrystalline thin films. Notably, as judged from PL measurements, the trap density within the single crystals is not in the range of 10^8^ to 10^11^ cm^−3^, but slightly lower than for the polycrystalline thin films on the order of 10^15^ cm^−3^. Furthermore, we illustrate the role of diffusion in time-resolved PL measurements in single crystals that strongly complicate the interpretation of early time kinetic data. Significant work is now required to consolidate optical spectroscopic and electronic measurements. We are currently undertaking detailed electronic measurements and theoretical modeling of single crystals in order to understand these phenomena, including the influence of mobile ionic species. Our findings here already suggest that polycrystalline perovskite thin films are much closer to possessing ‘single-crystal-like’ optoelectronic properties than previously thought, and improving optoelectronic homogeneity between grains under solar excitation intensities, as well as improving the electronic contacts in devices, remain the primary criteria to delivering near perfect thin films for photovoltaics and other optoelectronic applications.

## Methods

### Materials

DMF, PbBr_2_, formic acid, chlorobenzene, methylamine solution (33% in ethanol), poly(methyl methacrylate) (PMMA) and PCBM (> 99.9%) were obtained from Sigma-Aldrich, CH_3_NH_3_Br from Dyesol and spiro-MeOTAD from Luminescence Technology. Corp. For the preparation of thin films, PbBr_2_ from TCI was used. All chemicals were used as received without further purification.

### MAPbBr_3_ single crystal and thin-film samples preparation

The synthesis followed a rapid growth route at high temperature^34^. We prepared 1 M solution of the bromide salts in dimethylformamide and then added 3 vol% of formic acid to the solution followed by a filtration with 0.45 µm filter. We then incubated 5 ml of the solution in a closed cap vial at 55 °C to produce some seed crystals (~500 μm scale). In another vial we put a cleaned Si wafer or glass slides at the bottom of the vial and poured fresh salt solutions into the vial. We then carefully put a seed crystal of MAPbBr_3_ on the substrate, closed the cap and incubated the solution at 55 °C. The seed crystal grew on the Si substrate and we collected it when it had the desired size.

The preparation of MAPbBr_3_ thin films followed the route developed by Noel et al^82^. Equimolar amounts of MABr and PbBr_2_ were added to neat acetonitrile and stirred until an orange dispersion of MAPbBr_3_ crystals was formed. Methylamine gas was bubbled into the dispersion until the orange crystals dissolved to give a clear, colourless solution. Glass substrates were cleaned via successive sonication in Hellmanex (2 vol% in deionized water), deionized water, acetone, ethanol and isopropanol. The substrates were then treated with O_2_ plasma for 10 min to remove any organic residues on the surface of the glass. A 0.5 M solution of MAPbBr_3_ was then spincoated onto the glass substrate in dry air at 2000 r.p.m. for 45 s, resulting in the formation of a smooth, orange perovskite layer. The films were subsequently annealed at 100˚C for 10 mins to remove any residual solvent. The thickness of the films is 300 nm as determined with a stylus profilometer (Dektak 150). Finally, the films were protected by spin-coating a thin layer of PMMA (5 mg ml^−1^ in chlorobenzene) on top of the perovskite.

### UV-vis absorption spectroscopy

Optical spectra in transmission and reflection mode were measured with a Varian Cary 300 UV–Vis spectrophotometer with an internally coupled integrating sphere.

### Ellipsometry

Spectroscopic ellipsometry was carried out using a J.A.Woollam RC2 ellipsometer. The wavelength range measured was 210-1690 nm at three different angles: 55°, 65° and 75°. Final fits to the data using the general oscillator functions were carried out for all the angles simultaneously with J.A.Woollam’s CompleteEase software. The fit quality was high as reflected in the mean squared error close to 2.

### Steady-state and Time-resolved PL spectroscopy

Steady-state and time-resolved photoluminescence measurements where performed with a photon counting system (Fluotime 300, PicoQuant GmbH). The samples were excited with a 398 nm laser diode (LDH-P-C-405, PicoQuant GmbH, pulse duration 40 ps). Emission spectra were measured in front illumination mode where emitted light is collected from the side where the laser beam excites the crystal or in back illumination where the light is collected from the rear of the crystal. The distance traveled by the emitted light through the sample is approximately *d*_eff_ *= d*/cos(*θ*) where *d* is the crystal thickness and *θ* the angle between the crystal and the incident laser beam (Supplementary Fig. 6). Time-resolved measurements were acquired with a 447 nm laser diode (LDH-P-C-450B, PicoQuant GmbH, pulse duration 68 ps) at an excitation fluences between 7 and 500 nJ cm^−2^. We estimate the excited volume to be larger than the volume defined strictly by the penetration depth at the excitation wavelength (*d*_1/e_≈80 nm), due to the fast diffusion of charges within the crystal. Thus, the initial carrier density is estimated to be *N*_0_≈0.15–10.7 10^16^ cm^−3^. For time-resolved emission spectra, the crystals were excited at a fluence of 260 nJ cm^-2^ (*N*_0_≈5.6·10^16^ cm^−3^) and decay traces were recorded with 5 nm wavelength intervals. The single crystals were kept in air during the measurements.

### Two-photon excitation photoluminescence spectroscopy

One-photon and two-photon excited PL spectra were measured with a modified Leica TCS SP5 microscope. For one photon excitation, we used a 488 nm continuous-wave argon-ion laser. For two-photon excitation, we used a mode-locked Ti:Sapphire laser (Mai Tai) producing 150 fs pulses at repetition rate of 80 MHz and a wavelength of 800 nm. Then the PL spectra were acquired by scanning wavelength as a function of focusing depth, detected by an avalanche photodiode (APD). A pinhole (100 µm diameter) was used to decrease the signal out of the focusing plane.

### Data availability

The data that support the findings of this study are available from the corresponding author upon request.

## Electronic supplementary material

Supplementary Information
