# Supplementary material for: Consolidation of the optoelectronic properties of CH3NH3PbBr3 perovskite single crystals
Source: Nat Commun. 2017 Sep 19;8:590. doi: 10.1038/s41467-017-00567-8 (PMC5605602; doi:10.1038/s41467-017-00567-8)
Supplement: Supplementary file 1 — Supplementary Information [file 41467_2017_567_MOESM1_ESM.pdf]

Description of Supplementary Files

File Name: Supplementary Information
Description: Supplementary Figures, Supplementary Table, Supplementary Discussion and Supplementary References

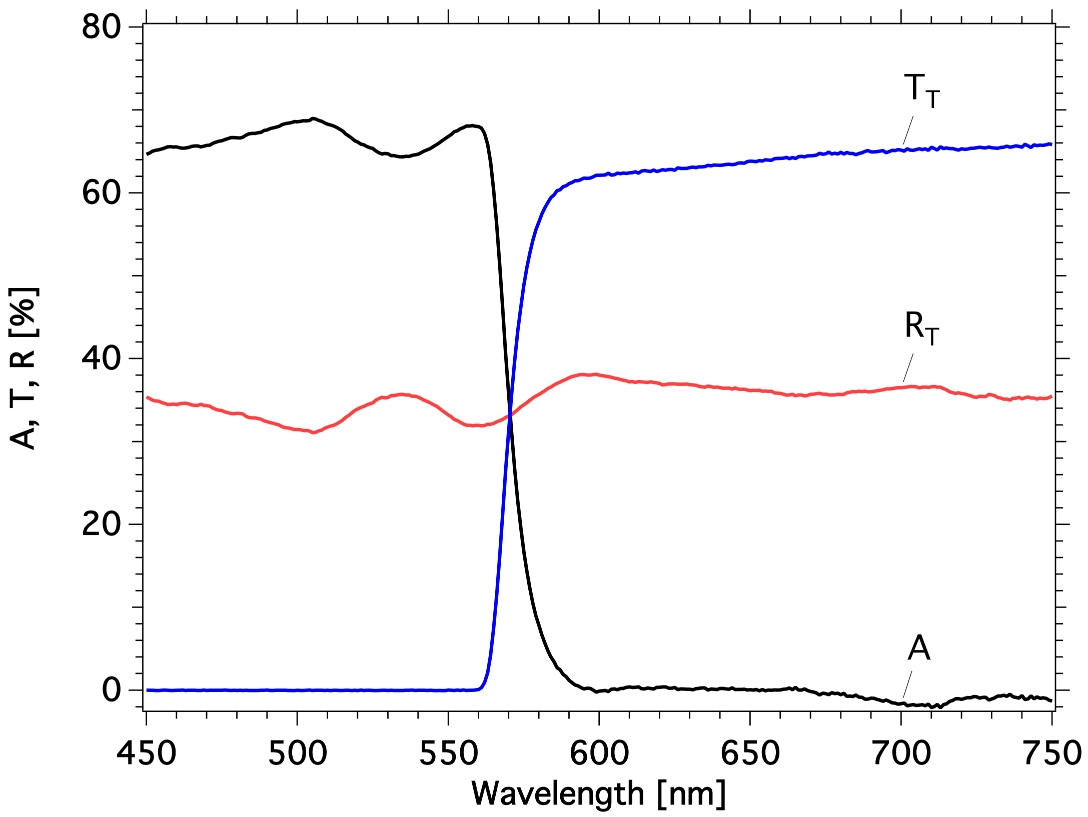

Supplementary Figure 1 | Total transmittance (T_T), total reflectance (R_T) and absorptance (A) of a $CH_3NH_3PbBr_3$ single crystal. Measured with a spectrometer equipped with an integrating sphere. The absorptance is calculated from $A = 1 - T_T - R_T$. We note that below 560 nm, no light is detected in transmission mode and the absorptance is no longer resolved.

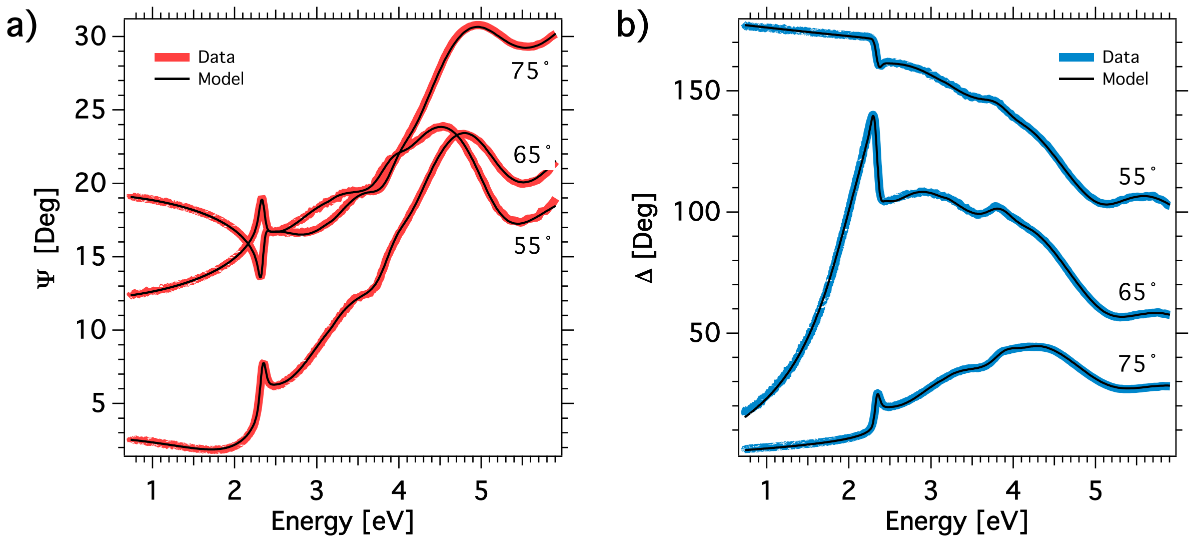

Supplementary Figure 2 | Spectroscopic ellipsometry data measured at different incident angles of a ~1 cm^2 $CH_3NH_3PbBr_3$ single crystal (~ 2 mm thick). (**a**) Psi values and (**b**) Delta values at different incidence angles. The model comprises of 1 Cody-Lorentz, 3 Gaussian and 1 Psemi-MO oscillator functions and uses an effective medium approximation to account for the surface roughness.

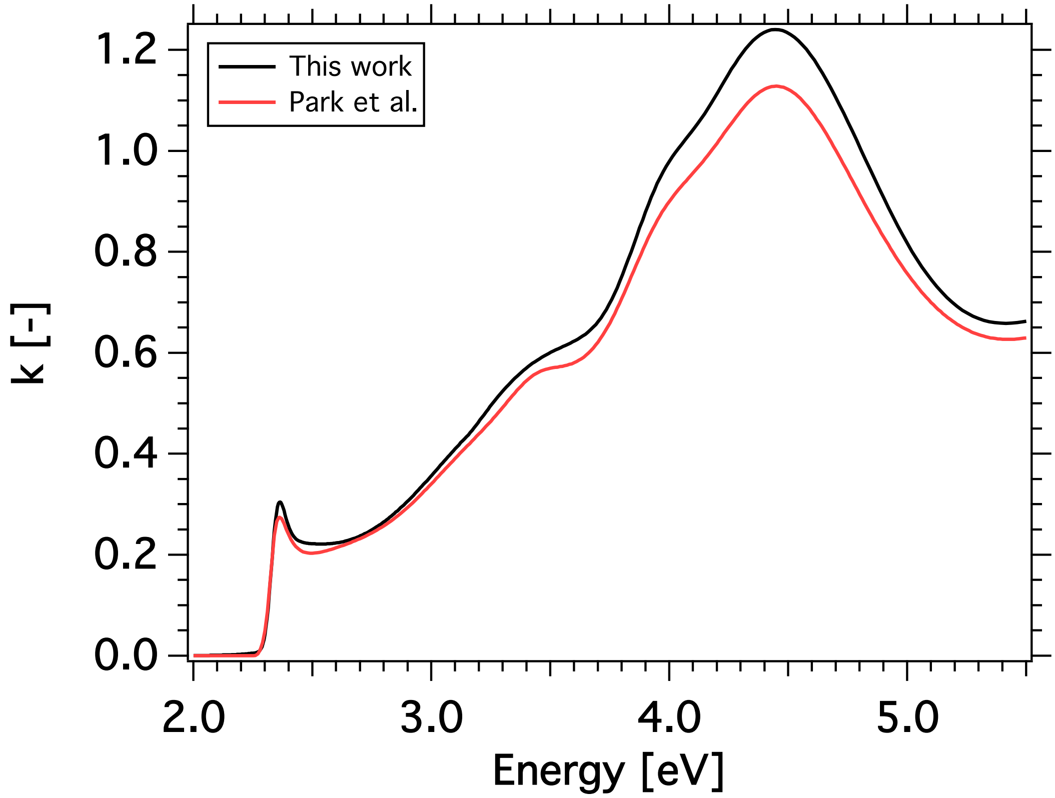

Supplementary Figure 3 | Comparison of the attenuation coefficient of $CH_3NH_3PbBr_3$ single crystals obtained from ellipsometry measurement in this work (black line) and by Park et al.[1] (red line)

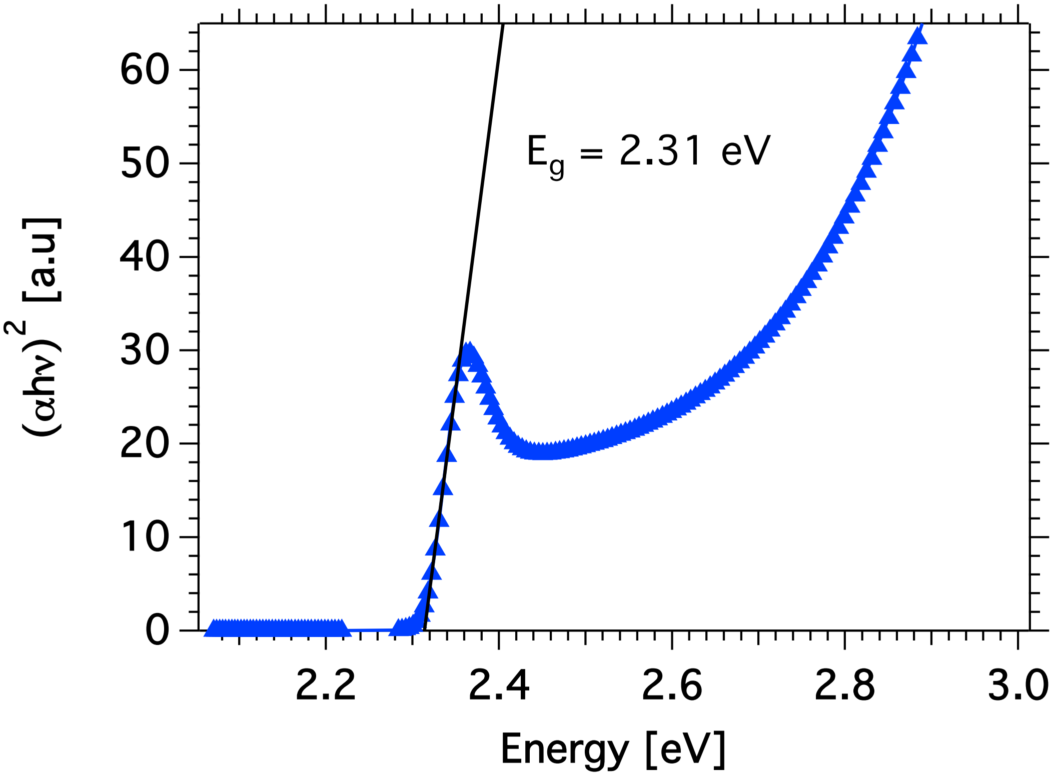

Supplementary Figure 4 | Estimation of the band gap energy of a single crystal of $CH_3NH_3PbBr_3$ with a Tauc plot for a direct bandgap material using the combined ellipsometry and transmission spectroscopy data. We note that the Tauc E_g is a slight underestimation of the electronic band gap due to the strong excitonic contribution to the band edge absorption.

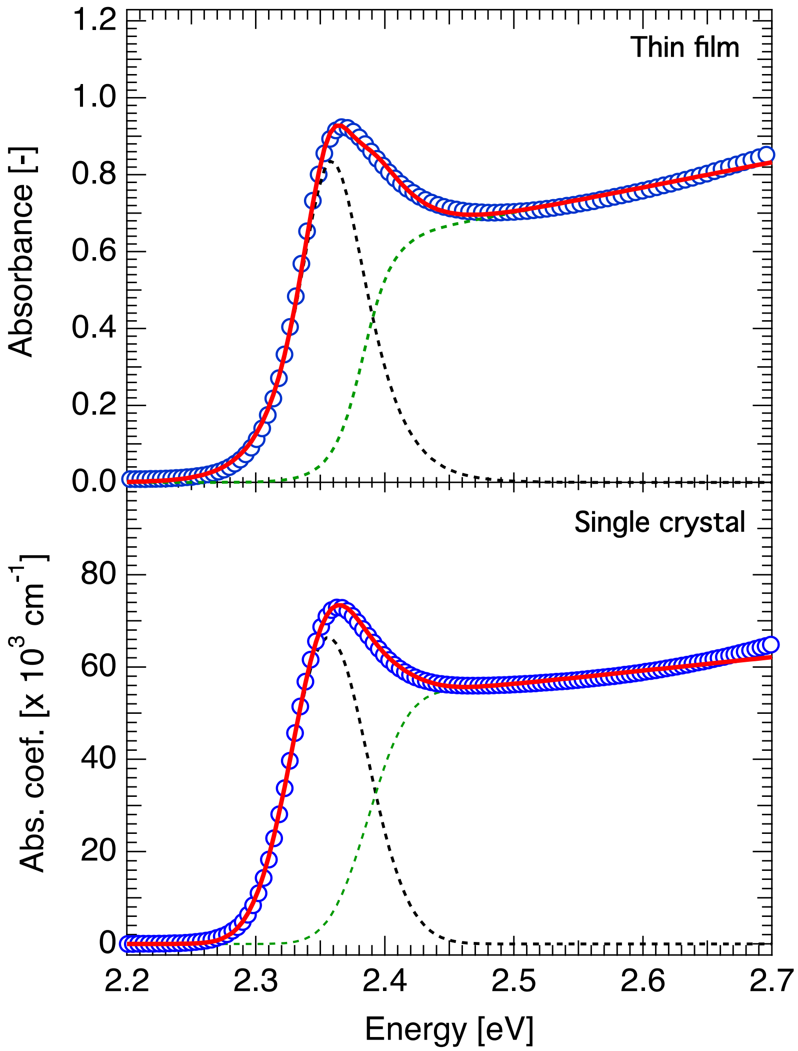

Supplementary Figure 5 | Fitting of the band edge region of the absorption spectrum of a single crystal of $CH_3NH_3PbBr_3$ with Elliott model using the formulation of Yang et al.[2] The best fit (red line) deviates from the experimental absorption spectrum (blue circles) for higher energies as only one band-to-band transition is taken into account. The model reveals an excitonic part (black dashed line) with a binding energy R_{ex} = 36 meV (thin film: 26 meV) and the direct absorption of the continuum sates (green dashed line) with a band gap energy E_g = 2.389 eV (thin film: 2.383 meV). The delta function for the excitonic transition and the step function for the continuum part are broadened by convolution with Gaussian functions with widths σ_{ex} = 28.2 meV (thin film: 14.7 meV) and σ_{cont} = 23.9 meV (thin film: 28.2 meV), respectively.

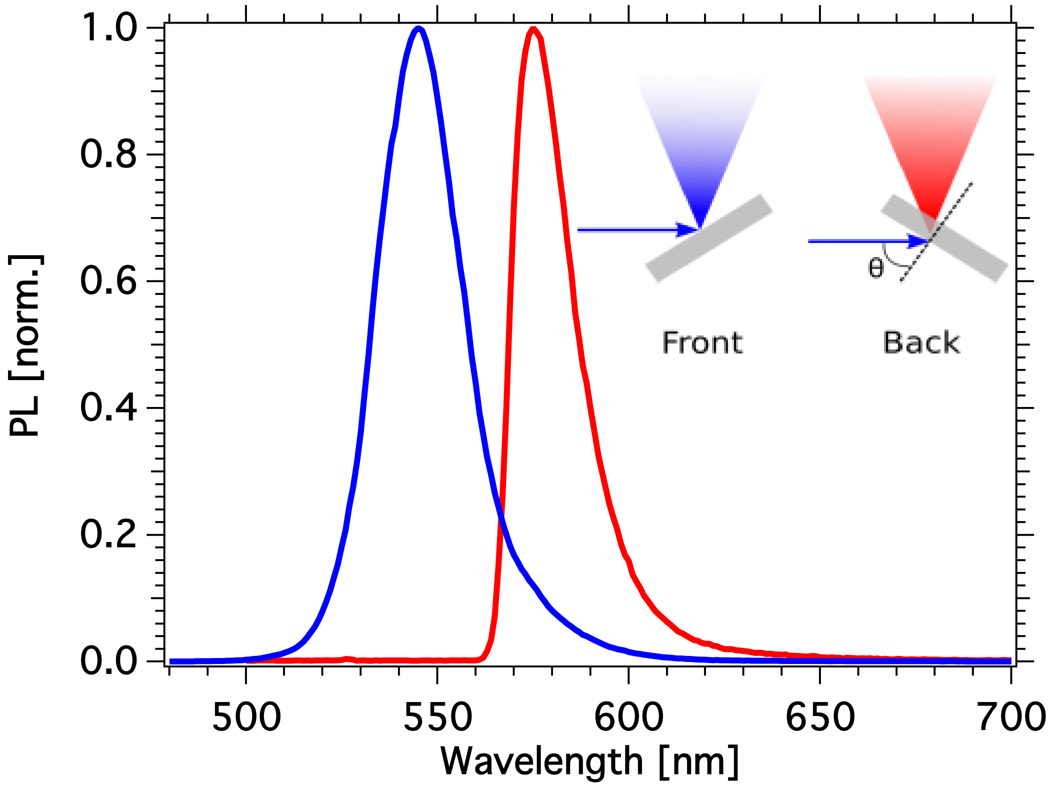

Supplementary Figure 6 | Normalised photoluminescence spectra of $CH_3NH_3PbBr_3$ collected with front illumination (blue line) and back illumination (red line). The full thickness of the crystal traveled by the emitted light is given by $d_{eff} = d/\cos(\phi)$ where d is the crystal thickness (d = 2.13 mm) and ϕ is the angle of the crystal with respect to the incident laser direction.

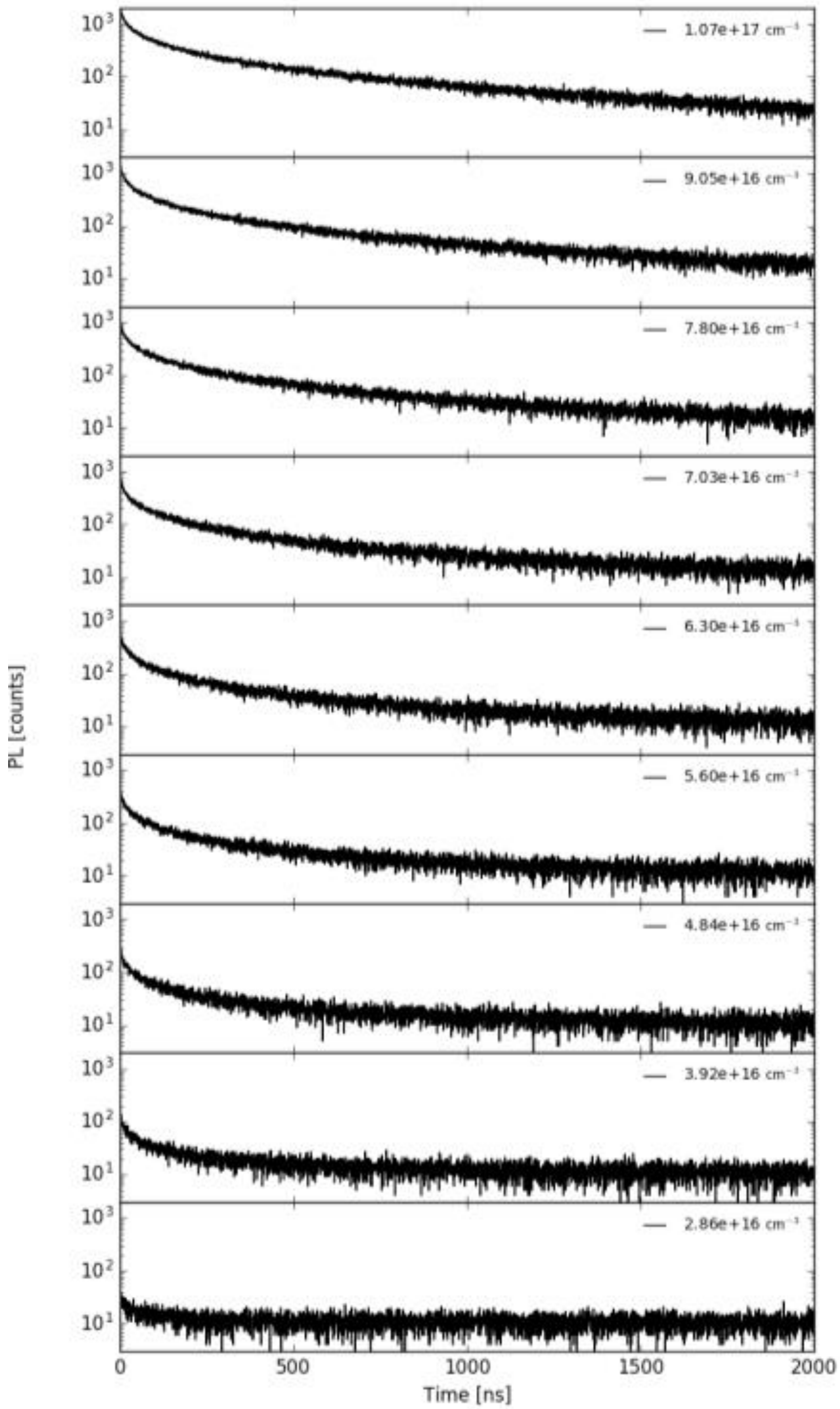

Supplementary Figure 7 | Photoluminescence decays monitored at 550 nm for a neat single crystal at increasing excitation densities (excitation wavelength: 447 nm). To estimate the carrier densities, we consider that 95% of the impinging laser light is absorbed within a thickness of 100 nm.

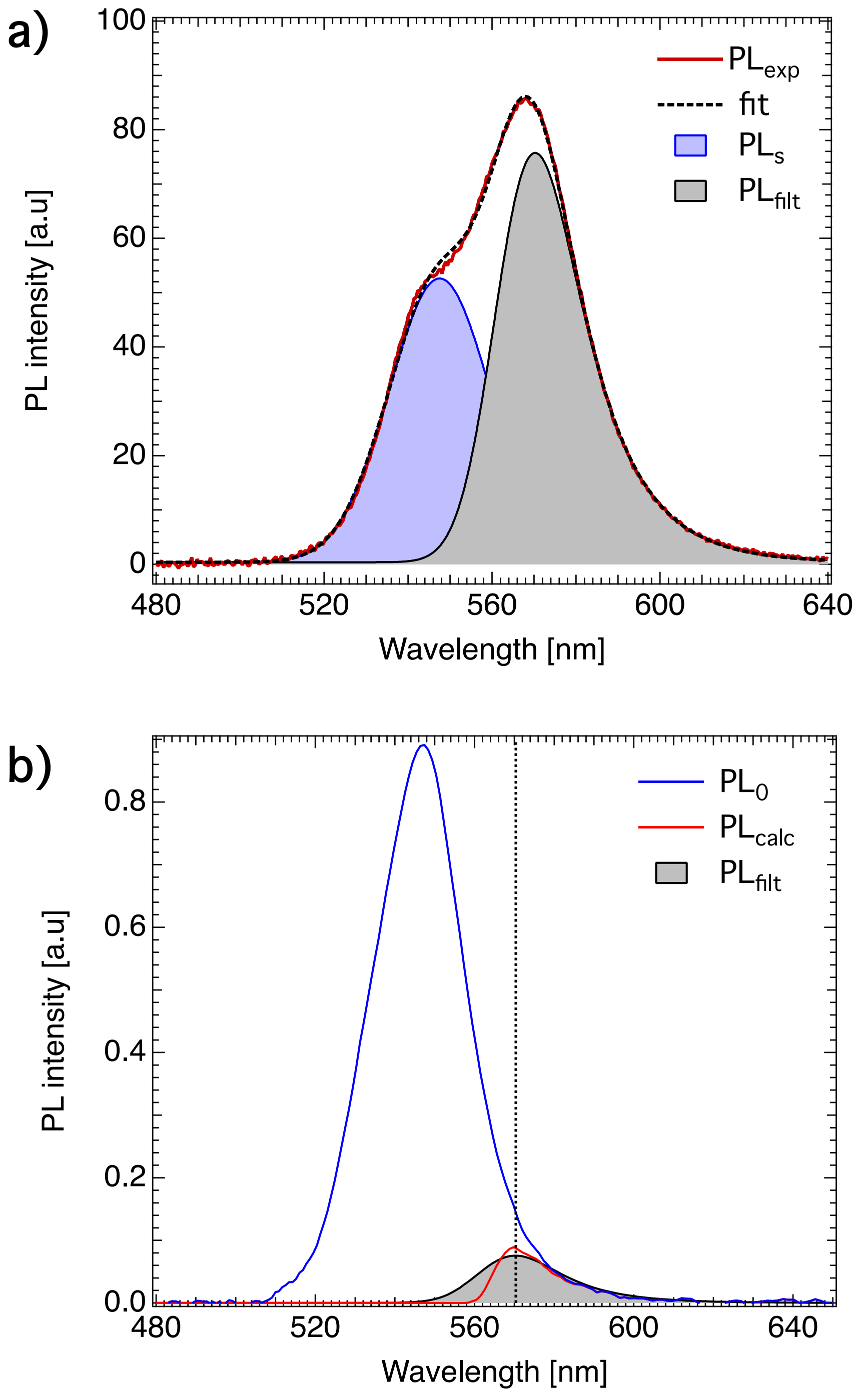

Supplementary Figure 8 | (**a**) Photoluminescence (PL) of a single crystal measured in an integration sphere (PL_{exp}) and fitted with two peaks representing emission from the surface (PL_{s}) and emission filtered by reabsorption through the crystal (PL_{filt}). (**b**) Simulation of the effect of filtering on the spectrum of a thin film (PL_{0}). PL_{calc} is the spectrum obtained by applying Beer-Lambert absorption on PL_{0}. See text for details.

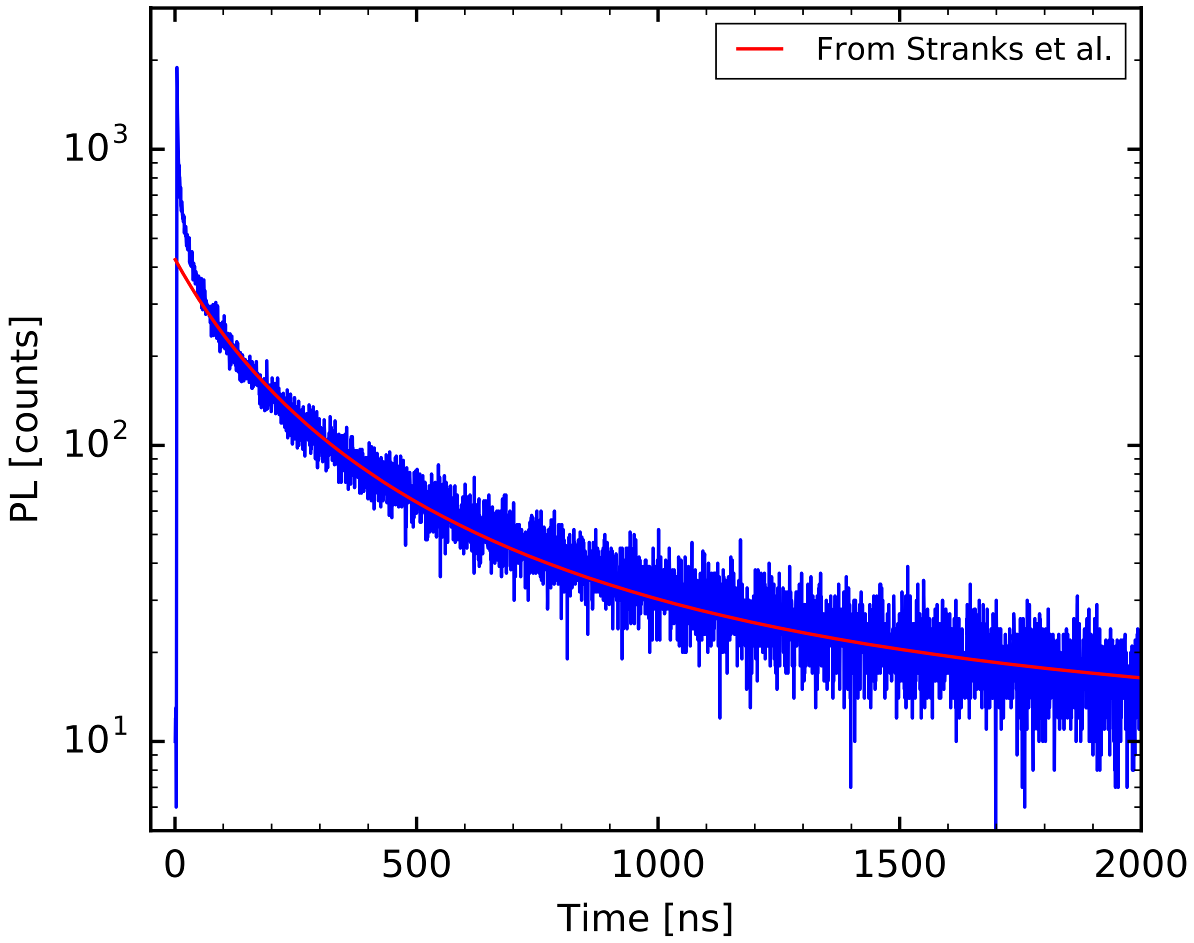

Supplementary Figure 9 | Evaluation of the fitting using the kinetic model from Stranks et al.[3]. Photoluminescence decay monitored at 550 nm for a neat single crystal (excitation: 447 nm, $N_0 \approx 7.8 \cdot 10^{16}$ cm^{-3}).

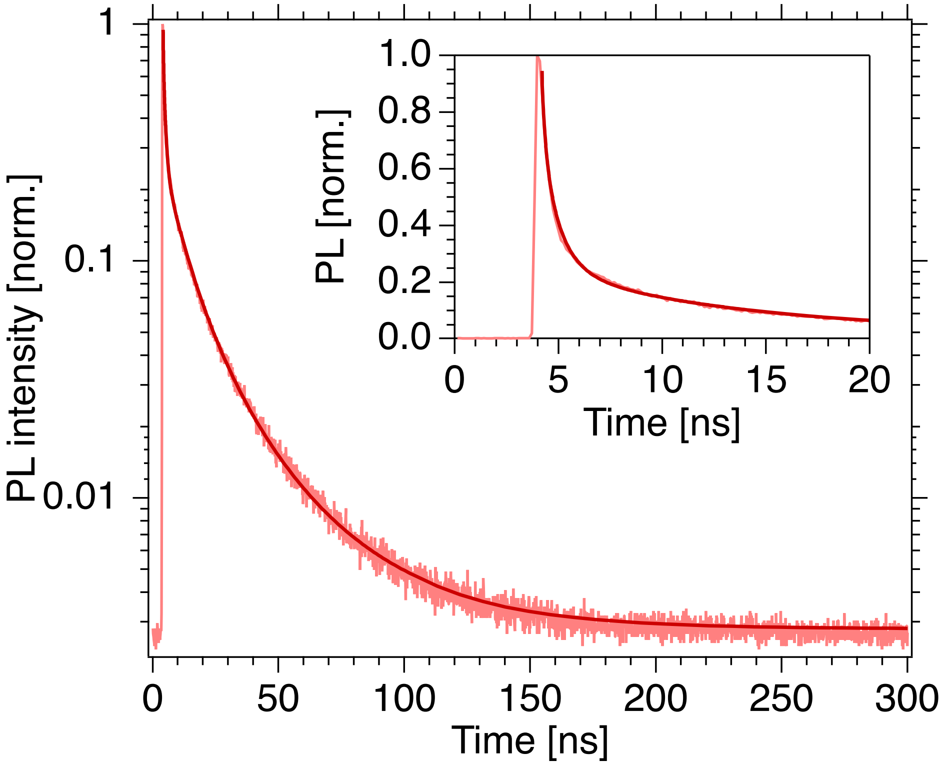

Supplementary Figure 10 | Numerical evaluation of Equation (2) for the photoluminescence decay of a $CH_3NH_3PbBr_3$ thin film (also shown in Figure 4) Fit parameters: μ_n = 1 $cm^2V^{-1}s^{-1}$, μ_{p} = 5 $cm^2V^{-1}s^{-1}$, k_2 = $1.2\cdot10^{-9}$ cm^3s^{-1}, $k_{1,n}$ = $1\cdot10^7$ s^{-1}, $k_{1,p}$ = $1\cdot10^4$ s^{-1}. Initial conditions: exponential carrier density profile with a penetration depth of 67 nm. See details of simulation below.

Supplementary Discussion

Estimation of the internal photoluminescence quantum efficiency

In Supplementary Figure 8 we show the photoluminescence (PL) spectrum of a small crystal of $MAPbBr_3$ (~1x2x0.2 mm^3) measured in an integrating sphere (excitation: 405 nm, 1 Wcm^{-2}). The measured external PL quantum efficiency is 6.4%. The spectrum shows two distinct peaks centred at 540 and 567 nm. We assign the high energy peak to light emitted from the surface of the crystal near the excitation volume. In contrast, the low energy peak is indicative of light being filtered by reabsorption within the bulk of the crystal. We exclude the presence of amplified spontaneous emission (ASE) since the shape of the PL spectrum does not change with the excitation fluence. Most of the light emitted inside the crystal is either trapped by total internal reflection or reabsorbed. We estimate the internal PL quantum efficiency by inversing the calculation of reabsorption presented in Figure 2b. First, we fit the measured PL spectrum to separate the contributions of surface and filtered emissions. The former is fitted with a Gaussian function whereas the latter is represented by the analytical convolution of a Gaussian with an exponential function to account for the asymmetry of the peak (see Figure 2c). As shown in Supplementary Figure 7a, these two peaks represent the experimental data well. To calculate the effect of filtering on the shape of the spectrum, we take as a starting point the PL spectrum of a thin film (PL_{TF}) of $MAPbBr_3$ deposited on a scattering substrate (frosted microscope slide). This sample shows minimal light trapping effects and is therefore typical of unaltered emission. We then simulate the effect of reabsorption and filtering by using Beer-Lambert absorption $I_{z,\lambda} = I_{0,\lambda}\exp(-\alpha_\lambda z)$ (see main text). We adjust z until the calculated peak maximum (PL_{calc}) coincides with the measured spectrum (Supplementary Figure 7b). Next, the original spectrum ($PL_0 \propto PL_{TF}$)) is scaled so that the calculate spectrum matches the height of the measured spectrum. Finally, the internal PLQE is estimated by taking the ratio of the area of the scaled peak and the measured peak: $PLQE_{int} = PLQE_{exp} \cdot [\text{area}(PL_0) / \text{area}(PL_{filt})]$. After subtraction of the light emitted directly from the surface, we estimate $PLQE_{int}$ = 67 %.

Simulation of photoluminescence decay

To simulate the photoluminescence decays at different intensities, we solve numerically Equation 2. As discussed in the main text the recombination rates contain first and second order components, therefore Equation 2 is rewritten for each carrier type as:

$$\frac{\partial n(z,t)}{\partial t} = G - D_n \frac{\partial^2 n(z,t)}{\partial z^2} - k_1^n n(z,t) - k_2 n^2(z,t)$$
$$\frac{\partial p(z,t)}{\partial t} = G - D_p \frac{\partial^2 p(z,t)}{\partial z^2} - k_1^p p(z,t) - k_2 p^2(z,t) \qquad \text{Eq. S1}$$

We use the Crank-Nicolson finite difference method to solve Equations S1. The initial conditions are given by an exponential distribution of carriers with a penetration depth of 67 nm as calculated from the absorption coefficient and the excitation fluence. The photoluminescence decay is given by:

$$I_{\mathrm{PL}}(t) = A \int_0^d B \cdot n(z,t) p(z,t) dz + C \qquad \text{Eq. S2}$$

Where *A* is a scaling factor, *B* the radiative recombination constant and *C* the experimental baseline. The carrier density profiles are integrated over the full crystal thickness *d*. The simulation parameters are given in Table S1. We note that to obtain good agreement with the experimental data we had to increase the *A* scaling factor for increasing initial carrier densities. This is consistent with trap-filling processes reducing non-radiative recombination at higher intensities.

Parameter	Symbol	Single crystal	Thin film
Crystal thickness	d	10 µm	300 nm
Total simulation time	t	10 µs	1 µs
Electron mobility	μ_n	10 $cm^2V^{-1}s^{-1}$	1 cm^2 $V^{-1}s^{-1}$
Hole mobility	μ_p	50 cm^2 $V^{-1}s^{-1}$	5 cm^2 $V^{-1}s^{-1}$
Bimolecular constant	k_2	$1 \cdot 10^{-10}$ cm^3s^{-1}	$1.2 \cdot 10^{-9}$ cm^3s^{-1}
Monomolecular constant (n)	$k_1^{(n)}$	$9 \cdot 10^5$ s^{-1}	$1 \cdot 10^7$ s^{-1}
Monomolecular constant (p)	$k_1^{(p)}$	$1 \cdot 10^3$ s^{-1}	$1 \cdot 10^4$ s^{-1}

Supplementary Table 1 | Summary of the parameters used to fit the decay curves in Figure 5 and Supplementary Fig. 10.

Supplementary References

1. Park, J.-S. *et al.* Electronic Structure and Optical Properties of α-$CH_3NH_3PbBr_3$ Perovskite Single Crystals. *J. Phys. Chem. Lett.* **6,** 4304–4308 (2015).

2. Yang, Y. *et al.* Low surface recombination velocity in solution-grown $CH_3NH_3PbBr_3$ perovskite single crystal. *Nat. Commun.* **6,** 7961 (2015).

3. Stranks, S. D. *et al.* Recombination Kinetics in Organic-Inorganic Perovskites: Excitons, Free Charge, and Subgap States. *Phys. Rev. Appl.* **2,** 34007 (2014).
